# A review of the selection process and decontamination methods with the use of face shields in UV phototherapy during the SARS‐CoV‐2 pandemic

**DOI:** 10.1002/ski2.97

**Published:** 2022-05-04

**Authors:** A. Granahan, J. McCavana, A. Lally, I. Morgan, S. Fitzgerald, B. Moriarty

**Affiliations:** ^1^ Department of Dermatology St. Vincent’s University Hospital Dublin Ireland; ^2^ Department of Medical Physics St. Vincent’s University Hospital Dublin Ireland; ^3^ Medical Microbiology and Infection Control St. Vincent’s University Hospital Dublin Ireland

## Abstract

Targeted ultraviolet (UV) phototherapy has been used in the management of a wide variety of dermatological clinical conditions including moderate to severe psoriasis unresponsive to topical therapies, vitiligo, severe atopic dermatitis and lymphoproliferative disorders. To date there are no uniform, standardised guidelines for the selection and decontamination process for UV personal protective equipment (PPE) and facial shields used in phototherapy. In the current climate, Coronavirus 2019 (COVID‐19) pandemic, standards regarding all decontamination and disinfection processes are under significant scrutiny. In terms of the UV‐PPE and facial shields used in phototherapy, careful disinfection procedures need to be implemented to ensure that the decontamination practice is effective enough to neutralise the virulent virus whilst maintaining maximal protection to the user from UV‐rays and safeguard the equipment from damage during the cleaning process. The aim of this report is to provide an evidence based review of the current and international practice standards guiding the selection, use and decontamination processes of UV facial shields in phototherapy. The complications and concerns that the COVID‐19 pandemic has had on this practice is highlighted. As such, we performed a comprehensive evaluation of the literature to provide recommendations as to the most effective, time efficient and safest practices for disinfection and decontamination of UV facial shields used in phototherapy during these unprecedented times.

1

**What is already known about this topic?**

- Evidence and research is very limited in the area of Ultra‐violent(UV)‐personal protective equipment (PPE) selection in phototherapy. The range of face shields and visors available is vast, but despite this no guidelines have been published outlining the recommended specifications to consider in this process. In the era of COVID‐19, the literature has become populated with extensive and novel disinfection and decontamination practices for PPE. However, in phototherapy care consideration into this practice is required not only to ensure the eradication of the virulent virus to prevent spread but to ensure that UV protection standards of the PPE used, is not damaged during this process.

**What does this study add?**

- No standardised guidelines or protocols regarding the selection process or decontamination process of PPE or face shields used in phototherapy published to date. During the COVID‐19 pandemic, PPE preservation and decontamination processes underwent significant scrutiny. In phototherapy the PPE requires extreme care, as incorrect disinfection practices can result in structure damage to the equipment reducing UV protection to the user. We report a comprehensive review and guideline into the specifications and selection process involved in PPE and face shield selection in phototherapy in addition to outlining the most safe and effective methods of UV‐PPE decontamination, in the era of COVID‐19.

## 2 INTRODUCTION

UV‐radiation (UVR), commonly used for many dermatological conditions can lead to potentially damaging and harmful biological effects. As such, it is recommended that UV protective facial shields in combination with other forms of PPE be worn to protect both skin and eyes during treatment. There is a paucity in the literature guiding the selection process and safety specifications required for the PPE and facial shields worn in phototherapy. With a vast number of facial shields available, it is incumbent that all phototherapy departments are aware of the safety qualities and standards required to provide optimum protection for patients undergoing treatment to avoid unnecessary harm.

To date, there are no standardised protocols or guidelines regarding the decontamination methods used for UV facial shields or PPE in phototherapy. With the rapidly emergent nature of the novel Coronavirus Disease 2019 (COVID‐19) there has been a concerted effort to find viable means of conserving PPE, including disinfection after use. As the incidence of COVID‐19 exponentially increases so too has the demand for PPE causing concern with supply meeting demand. To manage this, the literature became populated with novel innovative methods of preserving PPE, with new disinfection and decontamination processes. However, the UV‐PPE used in phototherapy requires extra care and caution as incorrect disinfection practices can result in damage to the structural integrity of the equipment impacting its protective qualities.^1^, ^2^, ^3^ Thus far, most dermatological phototherapy units employ simple effective cleaning measures guided by local microbiology, infection control and medical physics departments. Such processes include the use of soap, water and soft micro‐fibre cloths or a variety of sterile antibacterial cleansing wipes. However, there has been concerns if these measures are sufficient to neutralise the COVID‐19 virus.

Herein, with this report we provide guidance for phototherapy units on the safety standards and specifications required of UV facial shields employed in phototherapy. Following a comprehensive evaluation of the literature, we divulge the optimum disinfection and decontamination practices used for UV facial shields with reference to the challenges generated by the COVID‐19 pandemic.

## 3 RESEARCH OBJECTIVES AND METHODS

An initial survey was undertaken of the practices and policies of our local phototherapy units, to determine the selection process and disinfection practices employed in terms of UV PPE, with reference to the COVID‐19 pandemic.

A comprehensive literature review was undertaken, to identify international standards, guidelines, and policies to compare with local practices. References identified though the Medline, PubMed, Embase and CENTRAL databases. Scientific studies and research reports in peer‐reviewed journals were identified using relevant medical subject headings (MeSH) and field codes defining our clinical question. In addition, data from policy studies and briefs published by organisations including the Central Disease Control (CDC), World Health Organisation (WHO) were complied.

The eligibility criteria used for our literature review process involved the ‘population, interventions, comparisons, outcomes and study type’ guideline. Our population cohort included all adults (over 16 years) undergoing UV phototherapy for dermatological skin conditions requiring the use of UV‐PPE face shields during the COVID‐19 pandemic period, dating January 2020 to September 2020. The intervention involved all decontamination practices, including chemical and physical disinfection processes. Comparisons were possible evaluating different disinfection methods and practices used in different healthcare facilities and research units. The outcome was disinfection efficacy, viral (COVID‐19) neutralisation and the maintenance of the UV‐PPE structural safety for the users. There were no restrictions placed on study type, owing to the limited data available on this subject matter.

## 4 RESULTS

### 4.1 Part 1: UV facial shield selection

A thorough evaluation of the current UV facial shield products available was performed, with reference to the various facial shield specifications and recommendations to consider in a selection process. The literature explores a number of factors to ruminate, including the manufacturing visor material, the comfort and fit, the UV waveband protection in addition to the anti‐fog, anti‐glare and reflection qualities and the compliance with international certification standards.^4^, ^5^, ^6^

#### 4.1.1 Manufacturing visor material

Typically, facial shield visors are composed of durable robust material like polycarbonate (PC), acetate, polyethylene‐terephthalate glycol (PETG) or less commonly steel or nylon mesh. Additionally, the visor is commonly treated with advanced coatings to offer anti‐glare, anti‐fog and anti‐scratch properties depending on the intended use or application of the facial shield.^4^, ^6^ PC is a natural UV filter that effortlessly absorbs harmful UVR providing superior resistance to heat, extremes of temperature and impact, as such it has become the material of choice for UV protective facial shields in phototherapy.^4^, ^7^, ^8^ Acetate offers a reasonable level of protection from temperature, heat and UV exposure, it is prided with superior visual clarity, with innate qualities of anti‐fog, glare and scratch when compared with PC. Facial shields manufactured from PETG are robust and durable with a lengthier shelf‐life owing to their ability to withstand a multitude of environmental stresses including repeated decontamination processes. However the UV wavelength protection offered by PETG (less than 340 nm) is considerably lower than that offered by other materials available^9^ (Table 1).

**TABLE 1:** Comparison of facial shield visor material

Visor features	Material used		
	Polycarbonate	Acetate	PETG
Heat resistance	***	***	*
Impact resistance	***	**	*
Chemical resistance	***	**	***
Optical quality	** (180°C)	*** (190°C)	* (170°C)
Scratch resistance	*	***	*
Approximate cost	Lowest	Highest	Moderate

*Note*: Measure of protection or quality offered by the material of the facial shield visor.

*Poor/low; **Good/moderate; ***Best/highest.

References^7^, ^27^, ^44^.

#### 4.1.2 Design and structure

The structural components of the facial shield involve the visor material as described, the frame mount and suspension system. Most phototherapy units advocate for a lightweight plastic frame with an adjustable mount for individual fit, with some manufacturers offering a detachable mechanism for ease of cleaning. It is recommended from the Centre of Disease Control and Preventions (CDC) that the dimensions of all UV facial shields should span beyond the full length of the patients face including chin and crown areas and extend to the lateral aspect of the ears at a minimum.^10^ The appreciation of this standard is crucial, particularly during the COVID‐19 pandemic where disposable facial shields such as Hybec’s Durham Medimask became an interesting consideration to help manage infection control. However, the lack of complete facial coverage with this cone shaped shield does not comply with the CDC guidance nor does it meet the UV protection transmission requirement set out by the British Association of Dermatologists (BAD). Thus, precluding its use in our phototherapy unit.

#### 4.1.3 UV protection

UVR as part of the electromagnetic radiation spectrum, displays wavelengths ranging from 100 to 400 nm, shorter than visible light and thus carrying more energy. At specific wavelengths, UVR can exert biological effects at molecular levels which can lead to observable clinical effects. UVR wavelengths are broadly subdivided into three specific bands: Ultraviolet‐A (UVA) ranging from 315 to 400 nm; Ultraviolet‐B (UVB) from 280 to 315 nm; and Ultraviolet‐C (UVC) 100–280 nm.^11^ UVR light penetration is critical in phototherapy, with UVB primarily acting at the epidermis and epidermo‐dermal junction and UVA, due to its longer wavelengths, able to penetrate through the epidermis significantly far into the dermis.^12^ The harmful effects of UVR are extensively reported in literature, ranging from mild adverse reactions including hyperpigmentation, erythema, xerosis and pruritus to longer term risks of photoaging and photo carcinogenesis.^13^, ^14^, ^15^, ^16^, ^17^ Unfortunately, there is no reported universal standard guiding the exposure limits of UVR in phototherapy for patients. The CDC outlines a broad recommendation to keep ocular and facial exposure as low as possible, to safeguard patients whilst ensuring the required level of treatment is delivered to the intended areas. The BAD and European international standards advise all PPE used in phototherapy to offer, at a minimum protection from UVR waveband dosages between 300 and 400 nm. The use of both facial shields in conjunction with UV protective eyewear or goggles is further recommended.^18^, ^19^, ^20^ The BAD have outlined guidance for the transmission limits of protective UV eyewear and shields in phototherapy. At wavelengths at 390 nm the transmission limit is reported at 10%, at 380 and 370 nm it is 5% and 2%, respectively and 1% at wavelengths shorter than 360 nm.^21^ Considering this, it has been demonstrated that the most protective facial shields meeting these requirements include the Oberon™ UVP803 and Centurion™ Contour both offering UV transmission protection up to 400 nm and the Honeywell™ UVEX Bionic offering 380 nm. The Durhman Hybecs’medimask and the Bolles’ facial shield whilst offering a suitably secure and patient‐reported comfortable fit it was revealed that neither comply with the BAD transmission requirements.^4^

#### 4.1.4 Certification

There are a variety of international certification awarded to UV PPE that meet and comply with specific standards. These qualifications have been published extensively by European committee for standardisation (CEN) awarding the European norm (EN) and the European economic area certification (CE) and by the American national standard institute (ANSI).^6^, ^22^ These standards, the CE, EN and ANSI test the mechanical strength, durability and resistance as well as safety of the relevant PPE against environmental insults, biohazards and high speed UVR particles.^6^, ^23^, ^24^, ^25^ Although achieving these certification marks is not an absolute requirement for PPE or facial shields, it is strongly recommended that all phototherapy departments ensure compliance with the relevant scale and the EN170 at a minimum.^25^ Herein, we can summarise that the UV facial shields should be composed of PC or acetate material with an adjustable and/or detachable head‐mount system. We recommend the use of facial shields with international accredited standards like CE, EN or ANSI and in compliance with the BAD guidelines, provide UV protection up to 400 nm (Tables 2 and 3).

**TABLE 2:** UV‐facial shield selection considerations in phototherapy

Facial shield selection considerations	
Visor material	PC: Best heat and impact resistance
	Acetate: Superior clarity and scratch resistance, chemical splash and impact protection
	Propionate: Superior impact protection, stronger more robust
	PETG: Economical options
Comfort and fit	Headband: Adjustable and flexible for circumference
	Top band: Added depth for stability
Anti‐fog coating	Visibility: Thin film of polymers and hydrogels prevent fogging at temperature extremes
	Tested under the European certification EN 166/168 standard
Anti‐scratch coating	Visibility: Durability and abrasion resistance
Anti‐reflective coating	Visibility: Dissipate heat and remove glare
UV protection	Wavelength range and maximal transmission limits (BAD recommendations)
	Below 360 nm →1%
	361–370 nm →2%
	371–380 nm →5%
	381–390 nm →10%
Care and maintenance	Durability and re‐worn
	Disinfection and decontamination processes
	Soap and water (temperature controlled)
	Soft cloths, simple up and down strokes (avoid scratches especially with the PC facial shields)
	Anti‐bacterial cleansing wipes—alternative option
	Caution: Some solvents damage PC
Certification	American national standards institute (ANSI)
	Masks marked with manufactures ID
	Outlined ANSI Z87.1 section Mark Z87: Basic impact, resisting impact from 1 inch steel ball dropped height of 50 inches Mark Z87 +: High impact, resist impact from 0.25 inch steel ball at a velocity of 300 feet per second (91.4 m/s)
	European standards (EN166) withstand impact from 6 mm steel ball at various speeds
	Mark A: 190 m/s
	Mark B: 120 m/s
	Mark C: 45 m/s
	Regulation (EU) 2016/424 of the European Parliament and of the Council of the European Union
	Structure and design
	Storage and purpose of use of PPE
	Relevant UV protection factor number marked on PPE
	CE marking and EU declaration of conformity must be affixed to each individual PPE
	European Union PPE Regulation 2018
	SI no. 136/2018
Price	Cost effective
	Single patient use, disposable
	Decontamination and disinfection process
	Storage

References^4^, ^5^, ^6^, ^10^, ^18^, ^19^, ^22^, ^23^, ^24^, ^25^, ^27^, ^35^, ^45^, ^46^, ^47^, ^48^, ^49^, ^50^, ^51^.

**TABLE 3:** Specification and safety standards of UV facial shields used in phototherapy

UV facial shield	Visor material	UVR wavelength range (nm)	Compliant with BAD transmission limits (TL)	Weight (g)	Compliance standards	Benefits	Drawbacks
Hybec™ Durham Medimask H983	Clear composite plastic film	180–374	No	NR	CE	Low cost, disposable, withstands disinfection process Easy assembly, no specific storage requirements	Lateral coverage suboptimal
							BAD and EU standards noncompliant
Oberon™ UVP803	PC	200–400	Yes	408.23	ANSI Z87.1	Wide visual field (dimensions of shield larger than others 432 × 210 mm), lightweight, comfortable Lens Tint: Clear, Lens coating: Anti‐fog, Scratch resistance	Careful storage requirement
							Specific disinfection process
Analytik Jena UVP™ UVC‐803	PC	254–*365 Longwave ‘blue haze’	No *Inadequate TL over 360	794	CE ANSI Z87.1 1986 NIOSH	Shortwave and longwave (Blue haze protection) Adjustable secure fit	Heavier than other facial shields—uncomfortable TL concern
Honeywell™ Bionic S8500 (uncoated) S8510 (anti‐fog/hardcoat)	PC (acetate option)	200–*380	Yes *TL concerns above 380	630	CE ANSI Z87.1–2010 EN ISO9001:2000 EN169, EN170, EN 166	Most durable and heat resistant, 100% dielectric (no metals parts), secure customised fit (with 2784 positions), Built in chin guard Lens coating: Options for anti‐fog, scratch resistance, Lens tint: multiple options	Careful storage requirement Specific disinfection process TL concern Bionic electric arc model does not meet BAD or EU standards
Bolle Safety™ B‐LINE BL20FAPI	PC	Range NR 99.9% UVA and UVB protection	No Inadequate TL all wavelengths >360	254	CE ASNI Z87.1 EN 166 EN 170	Lightweight, adjustable with tilting shield, low cost, Lens coating: anti‐fog scratch resistance	Careful storage requirement Specific disinfection process BAD and EU standards noncompliant
Bolle Safety™ Safety Sphere, 2C‐1.2	PC	Range NR 99.9% UVA and UVB protection	No Inadequate TL all wavelengths >360	355	CE ASNI Z87.1 EN166, EN 170	Wide 180° field of vision, adjustable structure High energy impact protection, extreme temperatures (−5°C and +55°C) Lens tinti: Clear, lens coating: anti‐fog scratch resistance	Bulky robust design, more suitable to industrial operations rather than UV protection, Cleaning and storage specific requirement BAD and EU standards noncompliant
Centurion S592 9″	Acetate	280–400	Yes	150‐180	CE EN 170 EN 166 (Impact F)	Lens tint: Clear Lens coating: Anti‐fog, anti‐glare and scratch resistance	Careful storage requirements: dark, cool environment (structural risk)
Centurion™ Contour X1 S810	PC	280–400	Yes	170	CE EN 170 EN 166 (impact B)	Lens tint: Clear, lens coating: optional	Careful storage requirements: Dark, cool environment (structural risk)
Sibille™ E24	PC	280–*380*	Yes * TL concerns above 380	NR	EN166, EN167, EN168, EN170	Lightweight compact, comfortable fit with soft sweat band Panoramic visibility Lifespan estimate 2 years (indoor) 1 year (outdoor) Lens tint: Clear Lens coating: Anti‐fog, anti‐glare, anti‐scratch	TL concern with wavelengths above 390
Neiko™ 53819A safety	PC	280–*370	Yes *TL concern above 370	119	ANSI Z87.1 EN166 EN170	Universal fit (front and back adjustments), low cost, lightweight, comfortable Lens tint: Clear	Issues withclarity of lens/visor TL concern with high wavelength use
Catu™ MO‐286	PC	200–* 370	Yes *TL concern above 370	400	CE ANSI Z87.1 EN 166, EN170	Stable and secure fit—flip up position option Lens tint: Clear, Lens coating: Anti‐fog, glare and mist	More industrial based—arc flash and short circuit electrical protection (class 1)
UVEX™ S‐20755	PC	200–400	Yes	590	CE ANSI Z87+, Z87.1 EN166 EN170	Secure fit—two mount options (mechanical levers or magnetic locks), contouring shape of shield—excellent visibility Lens tint: Clear, lens coating: Options of uncoated, anti‐fog, glare and mist	Industrial use, advised to be used as secondary protection Uncoated options easily damage Specific storage and disinfection requirements
Petzl™ Vizen A014AA00	PC	NR	NR	180	CE ANSI Z87.1 EN166 EN170	Attachable device for helmet or headpiece, Life span 5 years (indoor and outdoor use) Lens coating: Options uncoated, anti‐fog, glare and mist	Requires additional safety PPE to ensure protection (glasses and spectacles must be worn—For industrial use

Abbreviations: ANSI, American national standards institute; BAD, British Association of Dermatologist; CE, conformité européenne; EN, European norm; EU, European Union; ISO, International Organisation for Standardisation; NIOSH, National Institute for Occupational Safety and Health; NR, not recorded; PC, polycarbonate; UV, Ultraviolet; UVR, UV‐radiation.

### 4.2 Part 2: Decontamination methods

In the current COVID‐19 pandemic, concerns regarding the durability, reusability and decontamination practices of the UV facial shields in phototherapy became rising concern. Questioning the efficacy of current decontamination practices and the ability of the facial shields to withstand repeated cleaning processes, to ensure the eradication of the virulent virus whilst maintaining integrity of the shields structure.

#### 4.2.1 Soap and water

Disinfection and decontamination processes for UV facial shields are guided by the manufacturing company. This process can involve the use of a plain soap and water, an environmental protection agency (EPA) approved or similar disinfectant or cleanser or an isopropyl based alcohol disinfection agent.^26^, ^27^ These methods must then be reviewed and approved by local infection control and medical physics departments, to ensure complete eradication of potential pathogens whilst maintaining the structural integrity of the UV PPE.^19^, ^26^ However, the virulent COVID‐19 pathogen has highlighted concerns regarding the safest and most effective decontamination processes for UV facial shields.^10^, ^27^ Limited literature and studies have been published to date in this regard, with a significant paucity of structured guidelines for decontamination processes for UV facial shields outside the manufacturing companies guide.

Few investigative studies have reported favourable results regarding the use of soap and warm water as a decontamination process for PPE and UV facial shields. These studies outline methods of submerging the facial shield in warm soap water and using a gentle soft micro‐fibre cloth to wipe the surface clear. However, comparative analysis between these studies is confounded by the global differences of the variety of ingredients used in the soaps as well as the inconsistent techniques used in terms of water temperature and duration of submersion in addition to the diverse range of training facilities available to those performing the decontamination process.^27^, ^28^, ^29^, ^30^

Surface disinfectant solution, spray or wipes are often used for UV facial shield cleaning process. Cleansing products that contain ammonium or that are high alcohol content must be used with caution as may lead to irreversible damage to the structure of the facial shield compromising the UVR protection. Similarly, there is a reported risk with these products creating visible film or residue on the surface of the facial shield that if not rinsed or cleaned in a timely fashion, can impair visual quality.^19^, ^27^, ^30^ Time and duration of use with the accepted disinfectant spray or wipe is critical, with studies reporting a required wet‐contact time ranging from 30 s up to 4 min depending on the composition of the solution used.

There are very few studies analysing the effectivity of these techniques; soap, water or disinfection solution on UV facial shields to eradicate COVID‐19^27^, ^30^ (Table 4). An International Czech based laboratory, SYNLAB in conjunction with University of chemistry and technology in Prague conducted a study evaluating the most effective method of disinfection against COVID‐19 using the Czech manufactured ‘Prusa’ UV facial shield. Recommendations from this study suggest the use of hydrogen peroxide at 25% strength for 5 min for successful bacterial and viral elimination, sodium hypochlorite 0.01% for 2 min, WHO 75% Isopropanol (IPA) hand‐rub disinfection for 5 min. UVC focussed decontamination methods using wavelengths below 280 nm for 15 min also showed positive results, with complete elimination of all bacterial and viral pathogens. Interestingly, Autoclave (AC) disinfection techniques, both hot AC (temperatures adjusted to 120°C, pressure setting at 200°kPa) and cold AC (temperature set at 60°C) were not recommended leading to significant shield structural deformation. Similarly, ethanol‐based disinfection solutions with measured strengths of 81%–100% resulted in structural abnormalities of the facial shield and reduced protection against UVR based phototherapy.^30^, ^31^, ^32^

**TABLE 4:** Recommendations of disinfection process for facial shields by manufacturing companies

Disinfection and decontamination recommendations		
Facial shield manufacturer	Facial shield model	Disinfection and care
Hysec™ limited	Durham Medimask, H983	Warm water and soap
		Sani cleansing wipes
		Disposable—individual use only
Oberon™	UVP803	Damp soft cloth wipe away visible grit/debris—flush with room temperature tap water
		Air or tap dry with soft cloth
		Other options:
		‐ Isopropyl alcohol (lysol wipe)
		5 min wet‐contact
		‐ 2% Clorox Bleach (sodium hypochlorite NaOCL)
		2 min wet‐contact
Face shield		
Analytik Jena UVP™	UVC‐803	No specific instructions outlined
Honeywell™	Bionic	Mild detergent in warm water
	S8500 (uncoated)	Clean soft cloth wipe
	S8510 (anti‐fog/hardcoat)	Cautioned scratch risk
Bolle Safety™	B‐Line, BL20FAPISafety Sphere 2C‐1.2	Rinse with cold water to remove visible dust, debris
		Cleaning solution, impregnated wipes
		Micro‐fibre soft cloth to dry
		Avoid hot water
		Avoid solvents, detergent, alcohol based agents outside recommended cleaning products:
		B‐Clean Bolle products:
		Aqua formula
		Silicone and alcohol free
		Mix: 5‐Chloro‐2‐methyl‐2*H* –isothiazol‐3‐one and 2‐methyl‐2*H* –isothiazol‐3‐one (3:1)
Centurion™	Contour X1	
	S592 9″ and S810	
Sibille™	Flip up E24 PC	Rinse warm water (temperature 30° C max)
		Gentle soft microfiber cloth
		Air Dry (max temperature 30)
Neiko™	53819A safety face shield	No specific instructions outlined
Catu™	MO‐286	Simple use of soap and lukewarm water (soap type not specified)
		Emsurse for 5 min
		Gentle cloth (type not specified)
		Air dry
Petzl™	Vizen face shield A014AA00	Rinse warm water (temperature 30° Cel max)
		Gentle soft microfiber cloth
		Air Dry (max temperature 30)

References^5^, ^6^, ^7^, ^19^, ^24^, ^26^, ^27^, ^31^, ^44^, ^52^, ^53^

Hybec Limited, Hospital Lamp Supplies, Access: www.hybec.com

Oberion Company, Access: www.oberoncompany.com

Analytik Jena AG, Endress Hauser Company Access: www.uvp.vom

Honeywell International Inc., Access: www.honeywell.com; www.honeywellsafety.com

UVEX Arbeitsschutz Group, Access: www.uvex-safety.com

Bolle Safety. Access: www.bolle-safety.com

Centurion Group, Safety Products, Access: www.centuriongroup.co.uk

Catu SAS Company, Access: www.catuelec.com

UVEX Arbeitsschutz Group, Access: www.uvex-safety.com

Petzl Professional Group, Access: www.petzl.com

In early March 2020, the environmental protection agency (EPA) released the initial ‘List N’ of disinfectants for use against SARS‐CoV‐2. The qualifying germicidal, disposable Sani‐cloth wipes revealed a 3‐log reduction against COVID‐19 following a 1 min contact time. As such, offers a safe, effective and time‐efficient method of disinfection for UV facial shields. However, to avoid any surface scratches it is recommended to pre‐rinse or submerge the facial shield in water to dislodge any particulate matter or debris.^33^

#### 4.2.2 Vaporised hydrogen peroxide (VHP)

Vaporised hydrogen peroxide (VHP) is an environmentally safe and commonly used industrial and healthcare decontamination process used in the sterilisation of reusable metal and non‐metal devices as well as medical instruments and materials. The ease of generation of VHP at low controlled temperatures in addition to its rapid cleaning cycle (30–45 min) makes it a potential method of efficient facial shield decontamination. When compared with other sterilisation or decontamination practices including UV‐Germicidal Irradiation (UVGI), VHP is reported to disinfect all crevices and cornered areas of the facial shield exteriors, thus a more thorough and effective result.^32^, ^34^ However, concerns with regards to the structural risk of the mask and facial shield with repeated VHP decontamination cycles are reported. Countering this, early studies reported by Viscusi outline that N95 Facial Filtration Respirators(FFR) masks undergoing treatment with VHP with temperatures up to 80°C on a 55 min cycle exhibited slight tarnishing of the nosebands with no damage to the filtering capacity of the mask.^29^ Similarly, Bergman reports studies of N95 FFR masks withstanding repeated cycles (3–5) at 125 min each without any filter or structural degradation noted.^35^ However, the safety profile and non‐damaging effect of VHP was validated following the FDA funded laboratory study by the Batelle Memorial Institute. In this report, following 50 treatment cycles of VHP the filtration performance and fit of the FFR was unaffected.^36^

Amidst the ongoing pandemic, more recent studies have evaluated the effectivity of VHP in the disinfection of masks or facial shields contaminated with SARS‐CoV‐2. Kumar et al. report complete eradication of SARS‐CoV02 virus on N95 FFR following a 1 h treatment process involving 10 min if dehumidification, 3 min conditioning, 30 min of decontamination and 20 min of aeration. The peak VHP concentration recorded was 750ppm, with the FFR withstanding 10 cycles of this treatment without any compromise to mask performance or structure. Similarly, Smith et al report no functional degradation to the FFR following two cycles of VHP treatment and no recoverable or viable SARS‐CoV‐2 virus.^3^ Interestingly, Fischer reports that VHP when compared with other disinfection treatments, including heat, ethanol and UVGI that VHP offers the best combination of rapid inactivation of the SARS‐CoV‐2 virus and preservation of FFR.^37^

#### 4.2.3 UV Germicidal Irradiation (UVGI)

UVGI is another disinfection process that has been extensively explored throughout the COVID‐19 pandemic. UVGI uses wavelengths between 180 and 320 nm to disrupt DNA and RNA cross‐linking, preventing pathogenic replication. At this wavelength, the UVR dose is directly proportional to the inactivation and elimination of the surface pathogens.^28^, ^38^, ^39^ However, whilst UVGI can offer an effective method of sterilisation, once again issues regarding the risk of structural damage to the facial shields and UV filter have been a cause for concern. Several studies have been published addressing these matters, with damaging results to the filters and fit structure, largely relating to higher doses of UVGI used.^28^, ^29^, ^30^, ^35^, ^39^, ^40^, ^41^, ^42^ From these studies, 4.68 Jcm^−2^ of UVR is reported as the highest recommended dose for which no physical or structural damage to the facial shield is observed.^29^, ^42^ However, as UVR is cumulative, the challenge remains to determine a safe baseline for the number of decontamination cycles that each individual facial shield can withstand. Furthermore, there is no standard published on the amount or dosage of UVR required to inactivate or eliminate COVID‐19 to date. A small number of studies have investigated this and report a minimal dose of 1.32–3.20 mJcm^−2^, required to eliminate approximately 90% of single‐stranded‐RNA viruses, like COVID‐19 on gel media.^28^, ^42^, ^43^ Innovative methods to deliver UVGI including the use of Biosafety Cabinets from Idle University Laboratories was explored in one study from the Cleveland Clinic. Effective decontamination was achieved by placing the facial shields into the biosafety cabinets. This process recommends a UVR dose for each facial shield of 1mWcm^−2^ for 20 min on each side. Whilst a promising and convenient disinfection method is outlined, the dose of UVR at different levels within biosafety cabinet varied considerably thus stringent quality control and monitoring measures are required to ensure the safety of the shields is preserved.^41^

## 5 CONCLUSION

In the absence of a clear consensus on the specification requirements of facial shields we provide a comprehensive review into the features and qualities that should be taken into consideration for all facial shields that are to be used in a phototherapy unit.

The COVID‐19 pandemic has led to development of several novel, innovative disinfection processes for PPE, including the use of VHP and UVGI. Whilst remain promising techniques they are not, at present easily or readily available in most healthcare or phototherapy units. In addition, the financial weight associated with these systems, in terms of staffing and equipment resources, requiring continuous specialised operational training and maintenance is high. We recommend from this review, the use of simple but thorough disinfection with soap, water and soft micro‐fibre cloths or germicidal disposable ‘Sani‐Cloth’ wipes. Regular staff training, educational appraisals and techniques reviews guided by infection control, microbiology and medical physics departments, are required to ensure safety standards are maintained.

With respect to ongoing planning for COVID‐19 we propose a process whereby each patient will receive their own designated facial shields for the duration of their treatment process. Between therapy sessions, simple disinfection measures will be taken as outlined and storage will be on site in patient specific lockers and safety cabinets. Following the completion of the phototherapy course, each facial shield should then undergo a comprehensive decontamination process prior to use by the next individual.

Certainly, going forward the use of VHP or UVGI should be a consideration in all healthcare facilities as well as phototherapy departments as a potential long‐term comprehensive decontamination solution to the COVID‐19 pandemic. However, further research is required into the safety, efficacy and costing as well as the overall procedural approach and technique of these methods before formal introduction into clinical practice.

## CONFLICT OF INTEREST

The authors declare they have no conflicts of interest.

## AUTHOR CONTRIBUTIONS

**Aoife Granahan:** Conceptualization‐Equal; Data curation‐Lead; Investigation‐Lead; Methodology‐Lead; Project administration‐Lead; Writing – original draft‐Lead; Writing – review & editing‐Lead. **Jackie Mc Cavana:** Data curation‐Supporting; Formal analysis‐Supporting; Investigation‐Supporting; Supervision‐Equal; Validation‐Supporting; Writing – review & editing‐Supporting. **Aoife Lally:** Conceptualization‐Supporting; Investigation‐Supporting; Methodology‐Supporting; Supervision‐Equal; Validation‐Supporting; Writing – review & editing‐Equal. **Imelda Morgan:** Data curation‐Supporting; Formal analysis‐Supporting; Investigation‐Supporting; Supervision‐Supporting; Writing – review & editing‐Supporting. **Susan Fitzgerald:** Data curation‐Supporting; Investigation‐Supporting; Supervision‐Supporting; Writing – review & editing‐Supporting. **Blaithin Moriarty:** Conceptualization‐Equal; Data curation‐Supporting; Formal analysis‐Supporting; Investigation‐Supporting; Methodology‐Supporting; Supervision‐Lead; Validation‐Equal; Writing – original draft‐Supporting; Writing – review & editing‐Lead.

## Data Availability

Data openly available in a public repository that issues datasets with DOIs.
